# Genome-Wide Changes in Protein Translation Efficiency Are Associated with Autism

**DOI:** 10.1093/gbe/evy146

**Published:** 2018-07-07

**Authors:** Igor B Rogozin, E Michael Gertz, Pasha V Baranov, Eugenia Poliakov, Alejandro A Schaffer

**Affiliations:** 1 National Center for Biotechnology Information, National Library of Medicine, NIH, Bethesda, Maryland; 2 School of Biochemistry and Cell Biology, University College Cork, Cork, Ireland; 3 National Eye Institute, NIH, Laboratory of Retinal Cell and Molecular Biology, Bethesda, Maryland

**Keywords:** autism spectrum disorder, ribosome profiling, codon usage, expression, CpG dinucleotides, single nucleotide variant

## Abstract

We previously proposed that changes in the efficiency of protein translation are associated with autism spectrum disorders (ASDs). This hypothesis connects environmental factors and genetic factors because each can alter translation efficiency. For genetic factors, we previously tested our hypothesis using a small set of ASD-associated genes, a small set of ASD-associated variants, and a statistic to quantify by how much a single nucleotide variant (SNV) in a protein coding region changes translation speed. In this study, we confirm and extend our hypothesis using a published set of 1,800 autism quartets (parents, one affected child and one unaffected child) and genome-wide variants. Then, we extend the test statistic to combine translation efficiency with other possibly relevant variables: ribosome profiling data, presence/absence of CpG dinucleotides, and phylogenetic conservation. The inclusion of ribosome profiling abundances strengthens our results for male–male sibling pairs. The inclusion of CpG information strengthens our results for female–female pairs, giving an insight into the significant gender differences in autism incidence. By combining the single-variant test statistic for all variants in a gene, we obtain a single gene score to evaluate how well a gene distinguishes between affected and unaffected siblings. Using statistical methods, we compute gene sets that have some power to distinguish between affected and unaffected siblings by translation efficiency of gene variants. Pathway and enrichment analysis of those gene sets suggest the importance of Wnt signaling pathways, some other pathways related to cancer, ATP binding, and ATP-ase pathways in the etiology of ASDs.

## Introduction

Autism spectrum disorders (ASDs) are characterized by difficulties in social interaction, difficulties in communication, and repetitive behaviors (Geschwind and State 2015). There is a statistically significant comorbidity of ASDs with intellectual disability (ID), including in monogenic forms of ID such as fragile X syndrome (Darnell et al. 2011). Since the 1980s, the United States (Wingate et al. 2012; Wingate et al. 2014) and some other Western countries (Atladottir et al. 2015) have seen substantial increases in the prevalence of autism. Partly due to the increase in prevalence, research efforts to identify factors contributing to ASD have intensified. These efforts include the collection and sequencing of DNA samples from hundreds of families (Krumm et al. 2015). Recent ASD research efforts also include epidemiological studies of environmental toxins and immunological factors, and cohort studies on the effect of changes in diagnostic criteria (Ornoy et al. 2015).

This study is focused on reanalysis of the data set in (Krumm et al. 2015) to test possible hypotheses about genome-wide genetic mechanisms in ASD etiology. We undertake this reanalysis while acknolwedging that many nongenetic factors are relevant, and we next summarize some of these factors.

The classification of ASDs changed fundamentally between the DSM-IV guidelines and the DSM-V guidelines, which were published in 2013 (Ornoy et al. 2015). Furthermore, various states within the USA have in recent decades changed the rules according to which ASD subjects receive assistance in schools. Multi-site studies across the USA show differences by a factor of at least 4 between the lowest incidence site and the highest incidence site (Wingate et al. 2012; Wingate et al. 2014). In contrast, ASD incidence in Italy did not increase in recent decades as much as in the USA (Ferrante et al. 2015). The large variations in incidence and prevalence suggest that local regulations (Rzhetsky et al. 2014) and/or local environmental factors (Volk et al. 2011) are associated with ASD incidence. Some studies have concluded that changes in diagnostic criteria in the 1990s and 2000s contribute as much as 1/3 to increased incidence of ASDs, at least in California (Grether et al. 2009; Hertz-Picciotto and Delwiche 2009; Herbert 2010), but this conclusion is controversial (King and Bearman 2009).

Exposure to air pollutants include: fine particulates (Becerra et al. 2013; Raz et al. 2015; Talbott et al. 2015), coarse particulates (Kalkbrenner et al. 2015) and ozone, nitric oxide and nitrogen dioxide (Becerra et al. 2013) have been repeatedly associated with autism. Environmental liquids and solids associated with autism include arsenic (Dickerson et al. 2015), lead (Dickerson et al. 2015; Hill et al. 2015), mercury (Dickerson et al. 2015) and pesticides (Rossignol et al. 2014; Ornoy et al. 2015). Lead and manganese were also associated with autistic-like behavior in a mouse model (Hill et al. 2015). Other risk factors include maternal uses of pharmaceuticals such as selective serotonin reuptake inhibitors for depression, (Croen et al. 2011; Boukhris et al. 2016) and valproic acid for epilepsy (Roullet et al. 2013; Hill et al. 2015). The risk of valproic acid was highlighted by a genetic study showing that valproic acid disproportionately reduces the expression of genes implicated in ASD because of likely deleterious mutations (Takata et al. 2018).

Other environmental studies have considered the role of prenatal and perinatal factors. These are of interest since genetic studies have identified an enrichment for mutations in genes that play a role in fetal brain development (Sanders et al. 2015; de la Torre-Ubieta et al. 2016; Yuen et al. 2017). One possible prenatal factor is the usage of prenatal ultrasound (Webb et al. 2017), but the association of ultrasound with ASD has not be replicated, to our knowledge. Pregnant mothers having flu, being hospitalized for an infection, or being treated with some classes of antibiotics (e.g., penicillin) were all associated with an increased risk of ASD in the child (Atladottir et al. 2012). The association of ASD with maternal hospitalization for infection has been replicated (Lee et al. 2015). Mothers of autistic children have, on average, above normal levels of some important cytokines, such as interferon γ (Goines et al. 2011).

A possible role for maternal cytokine levels in the etiology of autism, suggests considering other immune system components. Autistic children show patterns immune dysregulation, such as reduced frequency of naïve CD4+ T cells (Ashwood et al. 2006) and higher density of microglia-neuron pairs in close proximity (Morgan et al. 2012). The possible roles of genetic and immune factors are intertwined since various studies have shown statistically significant nonrandom inheritance patterns in ASD families of alleles or haplotypes variants in the human leukocyte antigen (HLA) region on chromosome 6 (Johnson et al. 2009; Guerini et al. 2011). Furthermore, a study of gene expression in post mortem brains of autistic and control subjects found differential expression in a module of immune-related genes including markers for microglia (Voineagu et al. 2011).

Now, we return to genetic studies, since this is our focus. Large-scale DNA sequencing studies seeking genes contributing to ASDs have been partly justified by the discovery of monogenic forms of ASDs. In addition, heritability studies in twins that show a higher incidence of ASDs in monozygotic twins compared with dizygotic twins of the same gender (Hallmayer et al. 2011; Frazier et al. 2014; Nordenbæk et al. 2014; Sandin et al. 2014; Colvert et al. 2015) although there is considerable variation in the estimates due to variations in methods (Tick et al. 2016).

Genome-wide sequencing studies have identified dozens of genes with recurrent deleterious mutations in ASD. Some of the proteins encoded by these genes cluster either in signaling pathways or in protein–protein interaction networks more than would be expected by a chance (Ben-David and Shifman 2012; Li et al. 2014; Chang et al. 2015; Wen et al. 2016). However, it is hard to conceive of a primarily genetic mechanism by which inherited mutations could contribute substantially to the rapid rise of ASD incidence. Therefore, several sequencing studies have searched for de novo mutations in the subjects’ germline, but not in the parents’ germline (Iossifov et al. 2012; Neale et al. 2012; O'Roak et al. 2012; Sanders et al. 2012; Krumm et al. 2015). De novo mutations from earlier generations can manifest as inherited mutations in new ASD patients if the de novo mutations arose in females, who have a lower penetrance as compared with males (Ronemus et al. 2014). De novo mutations could contribute to increased incidence for at least two reasons. First, the median parental age in the USA and other countries has been increasing, and the frequency of de novo mutations increases with parental age (Iossifov et al. 2012). Second, environmental toxins may exert their effects partly by increasing the mutation rate of gametes. The studies of de novo mutations found a statistically significant, increase in the rate of de novo likely deleterious mutations in ASD subjects compared with controls. Estimates of the contribution of de novo mutations to autism etiology range from a few percent (Gaugler et al. 2014) to over 40% (Ronemus et al. 2014). To put our work in context, consider that many mutations observed de novo in ASD subjects also occur in unaffected controls (Robinson et al. 2016). Combinations of rare and common variants can contribute to ASD susceptibility in the same individual via an additive oligogenic model (Chaste et al. 2017; Turner et al. 2017; Weiner et al. 2017).

An important contribution of the sequencing studies is that some of the data sets are available to other researchers who wish to explore alternative hypotheses about the etiology of ASDs. We do that here using part of the data set of (Krumm et al. 2015) that contains annotated exome sequencing data on 1,800 quartets (two parents, one affected child, and one unaffected child) and 592 trios (two parents and one affected child).

We previously proposed the hypothesis that perturbations that affect the efficiency of translation of mRNAs to proteins contribute to the etiology of ASDs (Poliakov et al. 2014). Henceforth, we use the term “protein translation” as shorthand for this fundamental biological process. We validated the genetic part of our hypothesis in a pilot study of 87 synonymous variants in 19 genes previously identified by other groups as ASD susceptibility genes (Poliakov et al. 2014). The hypothesis that protein translation is affected in ASD is reviewed in (de la Torre-Ubieta et al. 2016). It is supported by observations of likely deleterious mutations in genes such as *PTEN*, *TSC1*, and *TSC2*, as well as the long-established monogenic fragile X syndrome, which combines ASD and ID. In a mechanistic top-down approach, Darnell and colleagues (Darnell et al. 2011) showed that genes that encode mRNA targets of FMRP are significantly more frequently implicated in ASD than would be expected by chance.

In the current study, we test our hypothesis genome-wide using a much larger cohort (Krumm et al. 2015). In our principal analyses, we do not preselect the genes of interest in this study, but rather seek to identify by statistical methods those genes in which the pattern of translation-affecting synonymous variants differs between affected and unaffected siblings. In recognition that other groups have identified gene subsets of interest in ASDs, we repeated our principal analysis using those subsets of genes. We also analyze variants according to ribosome profiling occupancy, evolutionary conservation, and CpG context to evaluate whether single nucleotide variants (SNVs) present in affected individuals and absent in siblings (or vice versa) differ statistically by any of these characteristics pertinent to gene translation.

## Materials and Methods

### Ethics Statement

All the human subjects data in this study come from the National Database for Autism Research (NDAR) with approval by NDAR staff following their standard procedures. All data provided via NDAR are coded. More details are in the following subsections.

### Genotype Data

We obtained genotype data on 2,392 nuclear families with autism via NDAR. The primary data were associated with the study of (Krumm et al. 2015). These nuclear families consist of 1,800 families with two parents, one affected child, and one unaffected sibling, called “quartets” and 592 families with two parents and one affected child only, called “trios.” We used only the quartets. The primary data include the gender of the subjects. Due to the significantly higher incidence of ASDs in males compared with females, we partitioned the 1,800 quartets into four subsets, according to the gender of the affected sibling and the gender of the unaffected sibling.

### Reannotation and Filtering of Variants

The data provided by NDAR had been annotated with a mid-2014 version of snpEff (Cingolani et al. 2012) with respect to a May 2010 release of the human genome (Tychele Turner, personal communication to confirm the snpEff and genome versions). To get the data up to date, we used the newer SnpEff version 4.0 to reannotate all variants with respect to the build of the human genome known both as GRCh37.p13 and annotation release 105.

We initially considered all variants labeled by snpEff in the revised input as either “synonymous_variant” or “stop_retained_variant”, which are synonymous single-nucleotide variants (SNV) in the coding region of a gene. We integrated allele frequencies for European American and African American populations from the NHLBI Exome Sequencing Project (evs.gs.washington.edu/EVS). Most variants are annotated by SnpEff with a conservation score from PhyloP (Pollard et al. 2010). We performed the following filtering steps:

1. We removed variants whose snpEff annotation is inconsistent with GRCh37.p15 because of gene/transcript, the strand, or the codon.
2. We removed variants that could not be assigned to both a RefSeq Transcript starting with “NM” and an Ensembl transcript starting with “ENST.”
3. We removed all (variant, family) pairs for which the variant genotypes were missing in either sibling. Missing genotypes in the parents were allowed.
4. (recommended by the data suppliers) We removed all variants in segmental duplications (SegDup) and all variants with a high tandem repeats finder [TRF, (Benson 1999)] score above 50. These variants may not have been mapped reliably in the NDAR data.

For most of the analyses, we used SNVs such that the minimum recorded allele frequency is ≤10% or the maximum recorded frequency is ≥90%. We also included SNVs for which the allele frequencies are unknown because the SNVs with unknown frequencies are expected to have a rare minor allele. The second threshold is needed for the cases in which GRCh37.p13 has the minor allele as the reference. To test the robustness of our results, we redid some analyses using instead the threshold pairs (≤5%, ≥95%) and (≤15%, ≥85%). When AA, Aa, and aa genotypes, where a is a less common allele, were reported for the hemizygous parts of X and Y chromosomes in male subjects, we used A, a, and a genotypes, respectively.

### Targeted Gene Lists

We did the principal analyses genome-wide. Some analyses were repeated on lists of genes suggested by other groups to be implicated in autism. The SFARI Gene list is an evolving online database designed to permit quick entrée into the genetics of autism, and to help researchers track the ever-expanding genetic risk factors that emerge in the literature (https://gene.sfari.org/; last accessed March 14, 2017). A recent paper (Ji et al. 2016), presented lists of essential (EGenes) and nonessential (NEGenes) genes, based on the known roles of orthologs to these genes in mouse development combined with several other sources of information. Approximately half of all human protein coding genes were classified as either EGenes or NEGenes; we refer to all other human protein coding genes as “Unclassified.” For the SFARI, EGenes, and NEGenes lists, we used the gene symbols at www.hgnc.org to update the gene symbols in the published gene list to be consistent with the gene symbols in the NDAR data.

### Codon Usage

We obtained codon usage frequencies for brain-specific genes from (Semon et al. 2006), although the authors oppose the idea that natural selection shapes codon usage frequencies, as in (Poliakov et al. 2014) (supplementary table S1, Supplementary Material online). We also used codon usage frequencies for brain-specific genes from (Plotkin et al. 2004), codon frequencies averaged over all human genes (Warrington et al. 2000) and codon frequencies averaged over high confidence SFARI genes (https://www.sfari.org/resource/sfari-gene/) to test the robustness of our results. Let F(x) denote the frequency among human genes (possibly restricted to brain-specific genes) of codon x= “*NNN*”, where “*N*” denotes a nucleotide. $F\left(x\right)$ is an average, not dependent on a specific gene or a specific position within a gene.

### Translation Shift Score

For each individual *p* and variant $v_{i}$ at position i within the coding region of each gene *g*, a shift was calculated,

$$S\left(p,v_{i}\right)=\frac{F\left(w_{i}\right)-F(v_{i})}{F\left(w_{i}\right)}.$$

When a variant is in the homozygous state, it is counted twice. The term for a single variant is the *translation shift score* for that variant. Similarly, for each gene, we calculated a shift score

$$S\left(p,g\right)=\sum_{i\in V(p,g)}S\left(p,v_{i}\right),$$

where V(p,g) are the variants observed in individual pin geneg, $v_{i}$ is the codon induced by variant allele at i, and $w_{i}$is the wild type codon at position of variant iin the gene, this measure was used for a gene selection procedure (the section “Regression and gene selection using LASSO”). The shifts are not weighted by any characteristics of the gene such as the length of the gene or whether the gene is expressed in the brain. The value of the function Fdepends solely on the nucleotides forming the codon, not on the codon’s position within the gene. It so happens that no individual in these data sets had two variants that affected the same codon. In principle, we would handle such a situation for genes by representing the variant codon *once* in the sum, rather than once per SNV. If the patient and the paired unaffected sibling have the same genotype, such cases were not included in the analysis.

We used the median value of $S(p,v_{i})$ (among all the SNVs, not grouped by genes) observed in individual pas a summary statistic for individual *p*, when comparing affected individuals to unaffected individuals. A simplified version of the translation shift score was also used instead of the median value: we analyzed sums of signs of the translation shift scores (POS and NEG) for each pair of affected and unaffected sibling, aggregated over all SNVs in either individual. We used this sum value as a measure of the overall shift in each individual for pairwise comparisons of affected siblings versus unaffected siblings.

We analyzed synonymous SNVs separately in addition to analyses of all SNVs because synonymous SNVs were the focus in our analysis study of 19 genes previously identified by other groups as ASD susceptibility genes (Poliakov et al. 2014). It should be noted that Poliakov et al. found that synonymous SNVs with large values of the translation shift score tend to be associated with ASD, the mean absolute value for the studied synonymous SNVs was 0.53 (Poliakov et al. 2014). Therefore, for all but the first set of analyses we ignored individual SNVs for which the absolute value of that specific SNVs translation shift score was <0.5. We did similar calculations for codon frequencies derived from brain-specific genes from (Plotkin et al. 2004) (mean absolute value 0.44), codon frequencies averaged over all human genes (Warrington et al. 2000) (mean absolute value 0.32), codon frequencies averaged over high confidence SFARI genes (mean absolute value 0.24) and codon frequencies averaged over the list of 19 ASD susceptibility genes studied in (Poliakov et al. 2014) (mean absolute value 0.49). When we changed codon frequencies, the lower/upper thresholds for the synonymous SNVs were adjusted to be 0.4/−0.4, 0.3/−0.3, 0.2/−0.2, and 0.5/−0.5, respectively.

A paired parametric Student *t*-test (two-sided) and a nonparametric Wilcoxon matched pair test (two-sided) (the STATISTICA 4.5 program) were used to analyze differences between affected and unaffected individuals.

### Analysis of Ribosome Profiling Data

Five data sets of ribosome profiling in various human tissues were downloaded from the GWIPS-viz browser at http://gwips.ucc.ie/; last accessed February 12, 2016 (Michel et al. 2014, 2015). The ribosome profiling data provide information on ribosome locations at all mRNAs in the cell and the density of ribosome footprints depends on both the frequency with which a corresponding open reading frame is translated and the time that the ribosome dwells at a given codon. These data sets are described in the supplementary table S2, Supplementary Material online. Each ribosome footprint was represented at a single coordinate corresponding to the ribosome A-site which was inferred with a fixed offset of 15 nucleotides from its 5′ end as in the GWIPS-viz browser. The densities of footprints at the A-site codons were averaged in a window of 61 nucleotides around each SNV position in the transcribed and spliced mRNA (±30 bases surrounding each SNV). The signed ribosome score for a variant is the footprint density multiplied by +1/−1, depending on the sign of the translation shift score for that variant. For example, at position 9020509 of human chromosome 12 a synonymous SNV was detected (AAC>AAG); the averaged density of footprints is 19.0 and the codon usage frequencies for AAC and AAG are 0.019 and 0.031, thus the sign is −1. For the above example, the signed ribosome score is −19. The SNV positions were used to define windows on which to collect data, but the actual genotypes at the SNV of the individuals sampled for ribosome profiling were not treated as a variable. We also analyzed ribosome profiling scores for SNV affected codons only (without taking into account the ±30 bases surrounding each SNV), in addition we used this codon-specific ribosome profiling scores multiplied by translation shift scores instead of +1/−1.

### Analysis of Conservation Scores

We used PhyloP sequence conservation values (Cooper et al. 2005) provided in the revised snpEff annotation of the NDAR data. The signed PhyloP score for a variant is its nominal PhyloP score multiplied by +1/−1 depending on the sign of the translation shift score. For example, at position 9020509 of human chromosome 12 a synonymous SNV was detected (AAC>AAG); the PhyloP value is 0.157 and the codon usage frequencies for AAC and AAG are 0.019 and 0.031, thus the sign is −1. For the above example, the signed ribosome score is −0.157. For each individual, *P*, we computed the median of the PhyloP scores for the SNVs in that individual. We used the mean of the median PhyloP values distribution in the same way as for the translation shift score.

### Shuffling Procedure for Ribosome Profiling and Conservation Scores

The signed ribosome and PhyloP scores for a variant are its nominal ribosome profiling/PhyloP scores, respectively, multiplied by +1/−1 depending on the sign of the translation shift score. This creates methodological problems because any excess of scores for ASD patients can be explained by the excess of SNVs with positive translation shift signs observed in ASD patients (table 1). To test potential effects of the sign of translation shift scores, 100 random permutations of ribosome profiling and conservation scores across analyzed sets of SNVs was performed. The fraction of permuted sets with one of two probabilities values (parametric or nonparametric test) smaller or equal to the observed probability values was recorded.

**Table 1:** The Number of Rare and Moderately Common SNVs (10% MAF threshold) that Have Positive (POS) and Negative (NEG) Values of the Translation Shift Score

Data Set	#Families	Affected		Unaffected		*P*_Fisher_
		POS	NEG	POS	NEG	
All SNVs						
**All**	1800	**1030212**	**806181**	**1030158**	**810095**	**0.0099**
Ma–Mu	744	417012	322569	415560	324143	0.0059
Ma–Fu	828	476502	378133	477754	379383	0.4136
**Fa–Mu**	105	**66226**	**50280**	**65581**	**50743**	**0.0118**
Fa–Fu	123	70472	55199	71263	55826	0.4949
All synonymous SNVs						
**All**	1800	**529118**	**306455**	**528653**	**308594**	**0.0073**
Ma–Mu	744	214452	124876	213574	124709	0.2934
**Ma–Fu**	828	**244620**	**141475**	**245292**	**143146**	**0.0283**
Fa–Mu	105	34065	19332	33543	19382	0.0798
**Fa–Fu**	123	**35981**	**20772**	**36244**	**21357**	**0.047**
Synonymous SNVs with absolute values of codon shift score ≥ 0.5						
**All**	1800	**281599**	**227668**	**280328**	**229926**	**1.5 × 10^−4^**
**Ma–Mu**	744	**114246**	**92353**	**113122**	**92696**	**0.0151**
**Ma–Fu**	828	**129876**	**105398**	**130293**	**106821**	**0.0408**
Fa–Mu	105	18310	14456	17840	14421	0.0687
**Fa–Fu**	123	**19167**	**15461**	**19073**	**15988**	**0.0059**
Synonymous SNVs with absolute values of codon shift score ≥ 0.5 and all non-synonymous SNVs						
**All**	1800	**794391**	**726998**	**791949**	**731629**	**2.5 × 10^−5^**
**Ma–Mu**	744	**321804**	**295470**	**319811**	**296927**	**0.0010**
**Ma–Fu**	828	**368458**	**336237**	**368168**	**338563**	**0.0114**
**Fa–Mu**	105	**50471**	**45403**	**49878**	**45681**	**0.0254**
Fa–Fu	123	53658	49888	54092	50458	0.3548

Note.—One-tail Fisher exact tests (http://www.langsrud.com/fisher.htm) were used to test whether SNVs in affected individuals tend to have relatively more SNVs with a positive shift than unaffected individuals. Ma–Mu is affected male-unaffected male siblings, Ma–Fu is affected male—unaffected female siblings, Fa–Mu is affected female—unaffected male siblings, Fa–Fu is affected female—unaffected female siblings. Significant deviations according to the Fisher exact test from the homogeneous 2 × 2 tables are bold and underlined.

### Analysis of CpG Dinucleotides and Methylation Status

We analyzed the fraction of SNVs in CpG dinucleotides (fCpG) in affected and unaffected individuals. We used mean of the medians of fCpG scores in the same way as we used the mean of the median translation shift score.

For analysis of methylation, two data sets for brain cells (Meissner et al. 2008) were downloaded from the University of California Santa Cruz site (Brain BC H11058N, http://genome.ucsc.edu/cgi-bin/hgFileUi? db=hg19&g=wgEncodeHaibMethylRrbs; last accessed May 17, 2018). Most CpG dinucleotides in the hg19 reference genome are characterized by the methylated/unmethylated read count and the methylation ratio (the number of methylated reads divided by the total number of reads overlapping this position and multiplied by 100). If either the C or G is variable, it could be either the major allele or the minor allele, as determined earlier in Materials and Methods. To obtain a methylation ratio *M*_i_ for each position, we averaged the methylation ratios from the two data sets. The role of methylated CpG dinucleotides in exons is not well understood (Neri et al. 2017). Thus, we used a simple measure of the potential impact of methylation. A methylation shift score Ms is computed for each SNV that creates or removes a CpG dinucleotide. For each SNV in a CpG dinucleotide that changes a minor allele in the reference to a major allele not in the reference at dinucleotide *i*, Ms = M_*i*_. For each SNV in a CpG dinucleotide that changes a major allele in the reference to a minor allele not in the reference, Ms = 100 − M_*i*_. We used the mean of the median Ms values distribution in the same way as for the translation shift score and the PhyloP scores.

### Regression and Gene Selection Using LASSO

We used the software package “glmnet” (Friedman et al. 2010), developed in the GNU R programming system (R2013), to analyze the shift data. The glmnet package applies a regression method related to LASSO [least absolute shrinkage and selection operator (Tibshirani 1996), but applied to a generalized linear model]. Input into the glmnet package is a sparse matrix of shift scores, with individuals represented as rows and genes represented as columns, and a response vector containing an entry for each sibling, with a value of 1 indicating that the individual is affected, and −1 indicating the individual is unaffected. Among the output of glmnet is a vector of coefficients, one for each gene column. A nonzero coefficient indicates that glmnet choose to use that gene in the generated regression function. LASSO, by design, attempts to return a vector of coefficients that contains many zero elements, and thus identify the features (here, genes) that have nonzero coefficient to be the most relevant features.

The glmnet algorithm computes LASSO-type regression coefficients using a generalized linear model (GLM) subject to two penalty parameters: α and λ. The continuous parameter λ coarsely controls the number of genes that are included in the model; larger values of λgenerally produce models using fewer genes. The parameter α controls the use of ridge-regression regularization in the model. A value of α=1represents no ridge regression, whereas α=0 causes glmnet to use ridge regression exclusively, whereas values of αbetween 0 and 1 reflect different relative weighting of linear and quadratic penalty terms.

For a linear model, glmnet is formally described as follows. If $\left(x_{i},y_{i}\right)$ are Nobservations, where the components of $x_{i}$are the ptranslations shifts for the individual iand $y_{i}$is the response, then one must find a scalar $\beta_{0}$and p-vector β that solve

$$\underset{{(\beta }_{0},\beta )}{\min}\left[\frac{1}{2N}\sum_{i=1}^{N}{\left(y_{i}-\beta_{0}-x_{i}^{T}\beta \right)}^{2}+\lambda P_{\alpha }(\beta )\right],$$

where

$$P_{\alpha }\left(\beta \right)=\frac{1}{2}\left(1-\alpha \right)\beta^{T}\beta +\alpha \sum_{j=1}^{p}\left|\beta_{j}\right|.$$

Glmnet extends the linear model to a generalized linear model, using techniques described in (Friedman et al. 2010). Glmnet may be run in a mode that, for fixed α, finds a value of λ that minimizes misclassification error that is observed in cross-validation. We cross-validated the 744 male matched pairs by creating 744 training sets that omit a single pair. For each of these training sets, glmnet generated a classifier and tested its ability to correctly classify the two omitted individuals. We use glment to perform similar cross-validation for the 123 female–female pairs.

The overall scheme used to generate the candidate gene set G is shown in the left-hand column of supplementary figure S1, Supplementary Material online. Before applying glmnet, we scaled the columns of shifts, each of which represents the values of a given gene, to have a standard deviation of one. We did not center the values. Scaling and centering affects the magnitude of the optimal coefficients, and so is often applied before calling a LASSO-type algorithm; it is done by default in glmnet. However, while we wish to suppress differences in the magnitude of the shifts between genes, as it is not clear that these magnitudes are comparable, we expect that the sign of the change is important. Thus, we do not center, as centering may change the sign of some elements.

For the 11 fixed values α=0.0,…,0.9,1, we had glmnet use cross-validation to generate optimal binomial generalized linear models. For each tested α, we recorded the optimal $\lambda =\lambda_{min}(\alpha )$, and the coefficients of the linear model, one for each gene, produced for that value of α and $\lambda_{min}(\alpha )$. The full set of candidate genes, G, is the set of genes at the optimal parameter pair (α,λ).

To test the sensitivity of the genes results to changes in the input parameters, we generated three additional gene sets—G_50%_, G_0.005_ and G_filterdd_—as shown in the right-hand column of supplementary figure S1, Supplementary Material online. First, for each gene in G, we recorded how many times that gene was chosen by one of the classifiers generated by cross-validation. For the male–male set, this number was between 0 and 744, for the female set it was between 0 and 123. The set G_50%_ was defined to be the subset of G that was chosen in at least half of the cross-validation tests. Finally, for the 11 tested values of α, found the subset G_0.005_ of G consisting of genes that had a coefficient with magnitude at least 0.005 in the classifier generated by at least one of the tested α. For the most cautious lists of genes, we took the intersection of G_50%_ and G_0.005_ to arrive at G_filtered._

### Functional Annotation of Generated Gene Lists

We used STRING (Szklarczyk et al. 2017) to annotate the gene lists generated by LASSO analysis and to discover clusters of functionally connected genes using the Markov Cluster Algorithm (MCL). For such clusters, STRING reports a Protein–Protein Interaction (PPI) enrichment *P* value, indicating whether the generated network has significantly more interactions that expected. The MCL algorithm was run with the inflation parameter 4.5.

We used a second tool, GeneCodis (Carmona-Saez et al. 2007), to probe gene list enrichment for GO ontology, KEGG Pathways and Panther Pathways.

Because the LASSO-derived list for male pairs was large (1,224 genes), we also examined its intersection with the union of downregulated (M12 and MOD1) or upregulated genes in ASD patients (M16 and MOD5) from gene expression studies (Voineagu et al. 2011; Gupta et al. 2014). Voineagu et al. derived expression data from microarray experiments (Voineagu et al. 2011), and Gupta et al. applied RNA-seq technology (Gupta et al. 2014). Because of the distinct characteristics of these two gene expression technologies, we used the union of the gene lists from the two studies.

## Results

We analyzed a data set of 1,800 quartets comprising a father, mother, and two siblings (one affected and one unaffected individual) obtained from NDAR (see Materials and Methods, subsection Genotype data). For each individual p and variant $v_{i}$ in a coding region we computed a shift score $S(p,v_{i})$ that is based on the relative frequency of the codon induced by the variant to the wild-type codon (see Materials and Methods, subsection Translation shift score).

We checked our hypothesis that the codon usage shift (translation shift score) of rare and moderately common SNVs is associated with autism (Poliakov et al. 2014) in the simplest way. SNV minor allele frequencies (MAF) were obtained from the NHLBI Exome Sequencing Project (evs.gs.washington.edu/EVS; see Materials and Methods, subsection Reannotation of variants). The total number of rare and moderately common SNVs (MAF ≤ 10%) that are different between affected and unaffected sibling was counted. We also counted the total number of SNVs with positive values of translation shift score (more frequent codon changed to less frequent codon) (POS shift, table 1). We compared these numbers with the number of SNVs (MAF ≤ 10%) with the translation shift score ≤ 0 (NEG Shift, table 1) using the Fisher 2x2 exact test. We find that affected individuals have a significantly higher proportion of SNVs with a positive shift score (table 1). As in a previous study of ASD families (Ji et al. 2016), we split the 1,800 families into four subsets: affected male-unaffected male pairs (Ma–Mu), affected male—unaffected female (Ma–Fu), affected female—unaffected male (Fa–Mu), affected female—unaffected female (Fa–Fu). We found that the Ma–Mu subset and the Fa–Mu subsets have significant differences between affected and unaffected individuals (*P* = 0.0059 and 0.0118, respectively, table 1).

We tested the robustness of this observation by using the alternative thresholds of 5% and 15% for moderately common SNVs instead of the baseline threshold of 10%. The same trend was observed for both thresholds (supplementary table S3, Supplementary Material online). The 15% threshold produced significant results for All (all families together) and for Ma–Mu and Fa–Mu categories (supplementary table S3, Supplementary Material online) because the number of variants included is larger than for the 5% or 10% thresholds. All these results suggested that although the difference between affected and unaffected individuals generally was not substantial, the statistical significance (table 1 and supplementary table S3, Supplementary Material online) warrants further investigation. The tests in the table 1 and the supplementary table S3, Supplementary Material online are one-tail tests because they are attempting to confirm our previous results; in contrast, tests of new hypotheses below are done as two-tail tests.

Next, we repeated the comparison, omitting any synonymous SNVs with translation shift score <0.5 (see Materials and Methods). The use of this threshold substantially improved the results of statistical analysis, the difference between affected and unaffected siblings became highly statistically significant (*P* = 0.00015, table 1) for synonymous SNVs and the whole data set of SNVs after exclusions of synonymous SNVs with small changes of the translation shift score (*P* = 0.000025, table 1). Thus, in all further analysis, we ignore synonymous SNVs with translation shift score <0.5, except for one test in which we changed the source of the codon frequencies (see Materials and Methods, subsection Codon usage) and in the analysis of CpG dinucleotides.

### Analysis of Translation Shift Scores

**Fig. 1.**
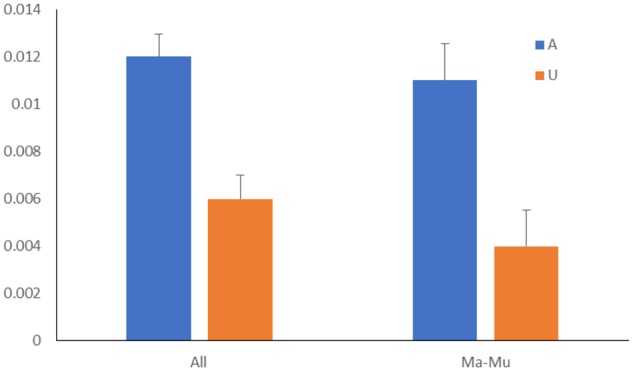
—Differences between affected and unaffected siblings using median translation shift scores. Scores were calculated in each individual for (*a*) all siblings and (*b*) affected male-unaffected male siblings.

For each individual, we used the median value of the translation shift score $S(p,v_{i})$ as a measure of the overall shift in each individual for pairwise comparisons of affected siblings versus unaffected siblings (see Materials and Methods). We found highly significant differences for the whole data set: the median for affected siblings was on average significantly larger than the median for unaffected siblings (fig. 1*a* and table 2). This difference is largely explained by a significant difference detected for the Ma–Mu data set (fig. 1*b*) although Ma–Fu and Fa–Mu sets also produced significant results (table 2). We also tested two thresholds for synonymous SNVs (0.25 and 0.75 instead of 0.5, supplementary table S4, Supplementary Material online). The results suggested that our analysis is robust with respect to the choice of the threshold (supplementary table S4, Supplementary Material online). Our analysis also appeared to be robust with respect to various codon usage tables (supplementary table S5, Supplementary Material online).

**Table 2:** Differences between Affected and Unaffected Siblings Using Median Translation Shift Scores Calculated in Each Individual

Data Set	Affected		Unaffected		Paired *t*-test (*P* value)	Paired Wilcoxon Z (*P* value)
	Mean	SD	Mean	SD		
All	0.012	0.041	0.006	0.042	**4.3 (0.00002)**	**4.3 (0.00002)**
Ma–Mu	0.011	0.042	0.004	0.042	**3.1 (0.00214)**	**3.1 (0.00175)**
Ma–Fu	0.014	0.039	0.009	0.041	**2.9 (0.00435)**	**2.7 (0.00635)**
Fa–Mu	0.021	0.043	0.009	0.037	**2.4 (0.01834)**	**2.3 (0.02119)**
Fa–Fu	−0.002	0.046	0.001	0.046	−0.6 (0.52963)	0.4 (0.69766)

Two-tailed paired tests were used to compare median values of translation shift scores calculated in each individual. Ma–Mu is affected male-unaffected male siblings, Ma–Fu is affected male—unaffected female siblings, Fa–Mu is affected female—unaffected male siblings, Fa–Fu is affected female—unaffected female siblings. Codon usage frequencies were taken from (Semon et al. 2006), as used in (Poliakov et al. 2014). Results for other codon usage sets (supplementary table S5, Supplementary Material online) are similar to the (Semon et al. 2006) codon usage data.

**Fig. 2.**
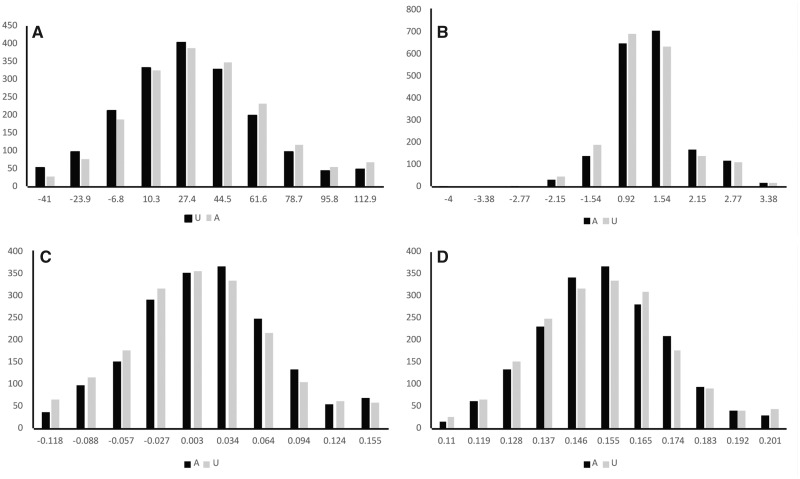
—Differences in scores between affected and unaffected siblings. Scores were computed using (*a*) sum of +1 and −1 indicating a positive or negative sign of translation shift scores calculated in each individual for all siblings, (*b*) median signed ribosome profiling scores for normal brain samples for all siblings (G14n, supplementary table S2, Supplementary Material online) calculated in each individual, (*c*) median conservation scores calculated in each individual for all siblings, (*d*) the fraction of SNVs in the CpG context calculated in each individual for all siblings.

We analyzed sums of signs of the translation shift scores (POS and NEG) for each pair of affected and unaffected sibling, aggregated over all SNVs in either individual, again ignoring SNVs with shift scores having magnitude <0.5. We used this sum value as a measure of the overall shift in each individual for pairwise comparisons of affected siblings versus unaffected siblings (see Materials and Methods for details). We found highly significant differences for the whole data set: the sum for affected siblings was significantly larger than the sum for unaffected siblings (fig. 2*a* and table 3). This significant difference is largely explained by significant differences detected for the Ma–Mu and Ma–Fu data sets (table 3). This observation cannot be explained by substantial differences in the number of SNVs for affected and unaffected siblings (at least the for the whole data set, Ma–Mu and Fa–Fu comparisons, supplementary table S6, Supplementary Material online).

**Table 3:** Differences between Affected and Unaffected Siblings Using Sum of +1 and −1 Indicating a Positive or Negative Sign of Translation Shift Scores Aggregated Over Each Individual

**Data Set**	**Affected**		**Unaffected**		**Paired *t*-test (*P* value)**	**Paired Wilcoxon Z (*P* value)**
	Mean	SD	Mean	SD		
All	40.87	33.51	35.63	33.64	**5.2 (<10^−6^)**	**5.0 (10^−6^)**
Ma–Mu	37.84	33.37	33.01	33.63	**3.0 (0.0025)**	**2.8 (0.0047)**
Ma–Fu	43.78	32.95	37.7	33.59	**4.2 (0.00004)**	**4.1 (0.00004)**
Fa–Mu	50.19	38.72	44.3	34.8	1.6 (0.1209)	1.6 (0.1201)
Fa–Fu	31.37	29.33	30.2	30.99	0.4 (0.6967)	0.3 (0.7968)

Note.—Two-tailed paired tests were used to compare the sum of the sign of the translation shift scores calculated in each individual. Ma–Mu is affected male-unaffected male siblings, Ma–Fu is affected male—unaffected female siblings, Fa–Mu is affected female—unaffected male siblings, Fa–Fu is affected female—unaffected female siblings. Codon usage frequencies were taken from (Semon et al. 2006).

We also did the test of translation shift scores on a subset of human genes known to be associated with autism, the SFARI (https://gene.sfari.org/) list (Materials and Methods, subsection Targeted gene lists). We analyzed translation shift scores for each pair “affected sibling versus unaffected sibling” for the SFARI list. Our analysis revealed marginally significant differences for the Ma–Mu subset: the median for affected males was significantly larger than the median for unaffected males for pairwise comparisons (supplementary table S7, Supplementary Material online). No significant differences were detected for other three subsets of families, or for all families combined.

A recent paper (Ji et al. 2016), which also reanalyzes published autism variant data, presented lists of essential and nonessential genes (EGenes and NEGenes, see Materials and Methods). The authors detected a significant excess of deleterious variants in EGenes in ASDs. We redid our main analysis specialized to the lists of EGenes and NEGenes, instead of all protein-coding genes. For the whole data set, significant differences were found when we analyzed translation shift scores for each pair “affected sibling versus unaffected sibling” (supplementary table S8, Supplementary Material online). Our analysis revealed highly significant differences for the All and Ma–Mu subsets for both EGenes and NEGenes and the two sets combined (supplementary table S8, Supplementary Material online). For the Ma–Fu subset, a marginally significant difference was found for nonessential genes whereas no significant difference was found for essential genes (supplementary table S8, Supplementary Material online). We also compared sets of merged EGenes and NEGenes versus all other human genes; a substantial difference was found between these two sets of genes (supplementary table S8, Supplementary Material online). This is consistent with the previous study of these two sets (Ji et al. 2016).

### Analysis of Ribosome Profiling

We also analyzed scores created by multiplying the sign of the translation shift score (+1 or −1) by the densities of footprints at A-site codon obtained from GWIPS (see Materials and Methods, subsection Analysis of ribosome profiling data). This *ribosome profiling score* was computed for each SNV (see Materials and Methods) for each pair “affected sibling versus unaffected sibling.” The intent of this analysis is to assign a weight to the +1/−1 scores in a manner that reflects a direct quantitative measurement of translation, thereby getting towards a possible mechanism. We used the median value of the ribosome profiling score for normal brain samples (Gonzalez et al. 2014) (supplementary table S9, Supplementary Material online) as a measure of the overall shift in each individual for pairwise comparisons of affected siblings versus unaffected siblings (see Materials and Methods for details). We observe a significant difference for the whole data set, for Ma–Mu subset and Ma–Fu subset: the median for affected siblings was significantly larger than the median for unaffected siblings (fig. 2*b* and table 4). No significant differences were detected for other two data sets. Results for other ribosome profiling data sets (supplementary table S9, Supplementary Material online) are like the normal brain samples (table 4).

**Table 4:** Differences between Affected and Unaffected Siblings Using Median Signed Ribosome Profiling Scores for Normal Brain Samples (G14n, supplementary table S2, Supplementary Material online) Calculated in Each Individual

Data Set	Affected		Unaffected		Paired *t*-test (*P* value)	Paired Wilcoxon Z (*P* value)
	Mean	SD	Mean	SD		
All	1.21	1.42	1.00	1.45	**4.5 (0.00001)**	4**.3 (0.00002)**
Ma–Mu	1.33	1.18	1.18	1.27	**2.7 (0.00714)**	**2.7 (0.00597)**
Ma–Fu	1.31	1.4	1.06	1.42	**3.8 (0.00011)**	**3.6 (0.00282)**
Fa–Mu	1.34	1.42	1.09	1.28	1.5 (0.14894)	1.4 (0.16684)
Fa–Fu	0.92	1.57	0.88	1.52	0.2 (0.84266)	0.3 (0.77661)

Note.—Two-tail tests were used to compare values of ribosome profiling scores calculated in each individual. Ma–Mu is affected male-unaffected male siblings, Ma–Fu is affected male—unaffected female siblings, Fa–Mu is affected female—unaffected male siblings, Fa–Fu is affected female—unaffected female siblings.

We used a permutation procedure (see Materials and Methods), to test whether the magnitude of the ribosome profiling score, rather than just the sign of the translation shift score, significantly distinguished autistic individuals from unaffected individuals. We found a significant and substantial impact of ribosome profiling scores on the outcome of the analysis, we did not observe any randomly shuffled sets with the probability values smaller or equal to the observed values for the “All” sets from brain tissues (normal G14n and tumor G14t, table 4 and supplementary table S9, Supplementary Material online). This suggests that the ribosome profiling score is a biologically important variable that allows a robust distinction of affected and unaffected individuals as large sets. We also analyzed ribosome profiling scores for SNV affected codons (without taking into account ±30 bases surrounding each SNV) and these codon-specific ribosome profiling scores multiplied by translation shift scores instead of +1/−1 (supplementary table S10, Supplementary Material online). Data sets of codon-specific ribosome profiling scores were much smaller compared with data sets of ribosome profiling scores used for the analysis above (averaged in a window of 61 nucleotides around each SNV position), however significant differences were observed for the whole data set, for Ma–Mu subset, Ma–Fu and Fa–Mu subsets (supplementary table S10, Supplementary Material online). The permutation procedure (see Materials and Methods) suggested that the observed significant differences are reliable at least for the “All” sets from the normal brain tissue (G14n).

### Analysis of Evolutionary Conservation

Most variants in the NDAR data were annotated with a conservation score from PhyloP (Pollard et al. 2010). We analyzed the PhyloP measure multiplied by the sign of the translation shift score (+1 or −1) for each SNV (see the Materials and Methods, subsection Analysis of conservation scores) for each pair “affected sibling versus unaffected sibling.” Our reasoning is that the ±1 score treats all variants above the 0.5 magnitude equally ignoring any evolutionary considerations; using the PhyloP score gives greater weight to variants at positions that are more conserved, where one might expect that a difference in translation efficiency could be more disruptive. We used the median value of the conservation score as a measure of the overall shift in each individual for pairwise comparisons of affected siblings versus unaffected siblings. We observed a significant difference for the whole data set and the Ma–Fu data set: the median for affected siblings was significantly larger than the median for unaffected siblings (fig. 2*c* and table 5). For the Ma–Mu data set, a marginally significant difference was found (table 5). No significant differences were detected for other two subsets.

**Table 5:** Differences between Affected and Unaffected Siblings Using Median Conservation Scores Calculated in Each Individual

Data Set	Affected		Unaffected		Paired *t*-test (*P* value)	Paired Wilcoxon Z (*P* value)
	Mean	SD	Mean	SD		
All	0.052	0.061	0.045	0.062	**3.7 (0.00027)**	**3.7 (0.00019)**
Ma–Mu	0.048	0.061	0.04	0.063	**2.5 (0.01299)**	**2.2 (0.03082)**
Ma–Fu	0.058	0.061	0.048	0.061	**3.6 (0.00032)**	**3.9 (0.00009)**
Fa–Mu	0.057	0.06	0.053	0.06	0.5 (0.60275)	0.1 (0.88814)
Fa–Fu	0.03	0.058	0.044	0.064	−1.7 (0.09702)	1.4 (0.16052)

Note.—Two-tail tests were used to compare values of signed PhyloP conservation scores calculated in each individual. Ma–Mu is affected male-unaffected male siblings, Ma–Fu is affected male—unaffected female siblings, Fa–Mu is affected female—unaffected male siblings, Fa–Fu is affected female—unaffected female siblings.

As with the ribosome profiling scores, we used a permutation procedure (see Materials and Methods) to evaluate whether the magnitudes of the conservation scores distinguish autistic individuals from nonautistic individuals. We did not find any significant impact of conservation scores on the outcome: for the “All” set the fraction of randomly shuffled sets with the probability values smaller or equal to the observed values (table 5) was 0.17. We conclude that the conservation scores do not allow any meaningful discrimination between affected and unaffected individuals. The observed significant differences between affected and unaffected siblings (table 5) are likely to be due indirectly to effects of the sign of translation shift scores (table 3).

### Analysis of SNVs in CG Dinucleotides and Potential Impact of Methylation

In our pilot study, we noticed that the frequency of variants in the CpG context is higher in the set of moderately common SNVs (MAF ≤ 10%) associated with ASD (Poliakov et al. 2014). The excess of mutations in CpG sites might reflect subtle differences in methylation, although that study (Poliakov et al. 2014) was certainly underpowered to detect genome-wide differences at CpG sites. The large-scale annotated nature of the NDAR data eliminates this potential problem.

We analyzed the fraction of SNVs in the CpG context for each pair “affected sibling versus unaffected sibling.” We observed a significant difference for the Fa–Fu data set: the fraction of SNVs in the CpG context for affected siblings was significantly larger than the corresponding fraction for unaffected siblings (table 6). No significant differences were detected for the other four subsets of families.

**Table 6:** Differences between Affected and Unaffected Siblings Using the Fraction of SNVs in the CpG Context and the Methylation Shift Score (Ms) Calculated in Each Individual

Data Set	Affected		Unaffected		Paired *t*-test (*P* value)	Paired Wilcoxon Z (*P* value)
	Mean	SD	Mean	SD		
Fraction of SNVs in the CpG context						
All	0.447	0.020	0.447	0.021	−0.2 (0.816)	0.6 (0.529)
Ma–Mu	0.448	0.02	0.448	0.022	−0.4 (0.718)	0.5 (0.618)
Ma–Fu	0.447	0.019	0.447	0.020	−0.7 (0.489)	1.1 (0.277)
Fa–Mu	0.448	0.019	0.449	0.018	−0.5 (0.621)	0.5 (0.644)
Fa–Fu	0.447	0.021	0.442	0.028	**2.0 (0.047)**	**2.0 (0.048)**
Methylation shift score						
All	83.6	24.7	81.7	24.6	**2.4 (0.015)**	**2.2 (0.026)**
Ma–Mu	82.6	26.4	80.2	27.0	1.9 (0.062)	1.7 (0.081)
Ma–Fu	84.5	21.3	83.6	21.5	1.0 (0.312)	0.9 (0.352)
Fa–Mu	76.5	26.6	79.9	24.6	−1.0 (0.297)	0.9 (0.364)
Fa–Fu	88.7	31.3	80.0	27.3	**2.7 (0.009)**	**2.4 (0.015)**

Note.—Two-tail tests were used to compare fractions/methylation shift scores calculated in each individual. Ma–Mu is affected male-unaffected male siblings, Ma–Fu is affected male—unaffected female siblings, Fa–Mu is affected female—unaffected male siblings, Fa–Fu is affected female—unaffected female siblings.

We also studied a potential impact of methylation. The methylation shift score Ms (see Materials and Methods) for each pair “affected sibling versus unaffected sibling” was compared. A marginally significant difference for the all data set was observed: the Ms score for affected siblings was significantly larger than the corresponding score for unaffected siblings (table 6). We also observed a significant difference for the Fa–Fu data set: the Ms score for affected siblings was significantly larger than the corresponding score for unaffected siblings (table 6). No significant differences were detected for the other three subsets of families (table 6). The fraction of SNVs in the CpG context and the methylation shift score can be considered largely independent measures and thus we can combine the *P* values using Fisher’s method [as a formula, *P*_combined_ = *P*_1_*P*_2_(1 − ln(*P*_1_*P*_2_)), which can also be derived via a Chi-squared test with four degrees of freedom]. For the *t*-test, the two individual *P* values of 0.047 and 0.009 yield a combined *P* = 0.004; for the Wilcoxon test, the two individual *P* values of 0.048 and 0.015 yield a combined *P* = 0.006. These results strongly support biological importance of methylation in ASD although they should be interpreted with a caution taking into account potential issues with independence of variables.

### Lists of Genes Potentially Associated with ASD

We tried to identify dozens to hundreds of genes that contribute to the genome-wide differences presented above (e.g., tables 1, 2, and 3). This can be viewed as a feature selection problem in machine learning, for which we applied LASSO algorithm, as implemented by glmnet (Materials and Methods, subsection Regression and gene selection using LASSO). A key virtue of LASSO is that it tries to limit the number of features (here, genes) selected. Reasoning that mRNA abundance is a weak proxy for protein abundance, we also combined the LASSO selections with prior knowledge on differential gene expression in ASDs (Materials and Methods, subsection entitled Functional annotation of generated gene lists).

Because the results in tables 1, 2, and 3 differ by gender, we decided to study genes in the male–male and female–female patient-sibling pairs separately. For pairs studied, individuals were divided into two classes, affected (class = 1) and unaffected (class = −1). We ran LASSO using the sum of the signs of the translation shift score, for all SNVs having translation shift score ≥0.5, as the independent variable for each gene. The 1,224 genes selected by LASSO for the Ma–Mu pairs, along with the LASSO coefficients, are shown in supplementary table S11, Supplementary Material online. Similarly, the 113 genes chosen for the Fa–Fu pairs are shown in supplementary table S12, Supplementary Material online. We repeated the LASSO analysis using CpG scores (each CpG-containing SNV was assigned a value of 1, all other SNVs are 0’s). The 183 genes chosen using CpG scores with Fa–Fu pairs is show in supplementary table S13, Supplementary Material online. Consistent with the lack of association shown between CpG scores and Ma–Mu pairs (table 6), LASSO using CpG scores separated the Ma–Mu pairs poorly. Thus, Ma–Mu LASSO analysis using CpG scores is not shown.

Enrichment analysis of the male–male gene list showed numerous KEGG (33) and Panther (14) pathways enriched with SNVs per GeneCodis (supplementary table S14, Supplementary Material online). These pathways included the Wnt signaling (Kegg: 04310, 12 genes, *P* = 0.046) and Panther: P00057, 23 genes, *P* = 0.0041), Neuroactive ligand-receptor interaction (KEGG: 04080, 26 genes, *P* = 0.00044), pathways in cancer (KEGG: 05200, 30 genes, *P* = 0.00044), and small cell lung cancer (KEGG: 05222, 13 genes, *P* = 0.00057). Reported *P* values from GeneCodis are corrected for multiple testing, by GeneCodis, using the method of false discovery rate (FDR). STRING enrichment analysis (supplementary table S15, Supplementary Material online) identified enrichment of various GO Molecular functions: catalytic (355, *P* = 0.00835), hydrolase (180, *P* = 0.0027) and ATP-ase activity (32, *P* = 0.0356). At a higher level of GO Biological process, several processes were also significantly enriched: metabolic, primary metabolic, organic substance metabolic, and cellular metabolic processes.

The Wnt signaling pathway is a prevalent theme in our enrichment analysis. Recently, it was proposed to be central for proper development of neurons and mutations in these genes were shown to be high confidence or likely causative for autism (Caracci et al. 2016; Kwan et al. 2016). Analysis of male–male translation shift scores using GeneCodis and GO ontologies demonstrates highly significant enrichment in ATP-binding proteins (120, *P* = 1.26 × 10^−14^) and nucleotide binding proteins (155, *P* = 2.51 × 10^−15^) (supplementary table S14, Supplementary Material online). This theme is also consistent with another recent hypothesis about autism etiology, which implicates ATP as central stress signaling molecule in cell danger response (CDR) response (Naviaux 2014; Naviaux et al. 2013, 2017).

We did not expect relevant pathways to show up in enrichment analysis of the female–female list derived from translation shift scores because no statistical significance was shown in that analysis (tables 2 and 3). In fact, the enrichment analysis with GeneCodis identified three KEGG pathways (supplementary table S16, Supplementary Material online). Those pathways were: Ribosome biogenesis in eukaryotes, KEGG: 03008 (three genes, *P* = 0.035), Mineral absorption, KEGG: 04978, (three genes, *P* = 0.037); and p53 signaling pathway, KEGG: 04115, (three genes, *P* = 0.0041).

Modular enrichment analysis with GeneCodis of the LASSO-derived CpG female–female list (supplementary table S17, Supplementary Material online) demonstrated that the Wnt signaling pathway is enriched in SNVs associated with ASD patients [KEGG: 04310 (*P* = 0.014) and Panther: P00057 (*P* = 0.021)]. It was also shown that the basal cell carcinoma pathway KEGG: 05217 (four genes, *P* = 0.017)] as well as Neuroactive ligand-receptor interaction (KEGG: 04080, seven genes, *P* = 0.025) may be important for ASDs.

**Fig. 3.**
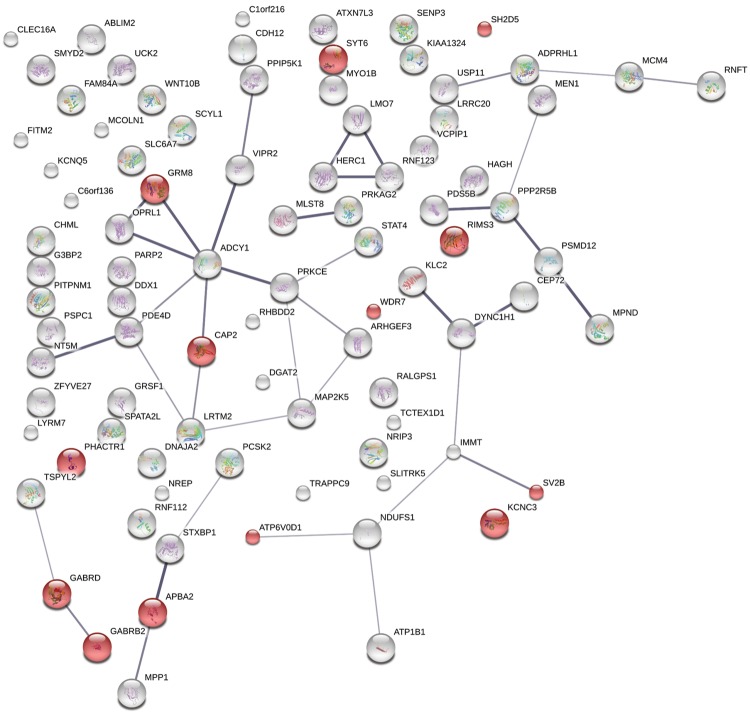
—Network of functionally connected genes. The network was reconstructed using the STRING program obtained by intersection of LASSO male–male list and downregulated modules in ASD patients as the input (Gupta et al. 2014; Voineagu et al. 2011). GO: 0045202 synapse-classified genes are shown in red.

We also examined the intersection of the LASSO male–male list (1,224 genes) and downregulated modules (M12 and MOD1) or upregulated genes modules in ASD patients (M16 and MOD5) (Voineagu et al. 2011; Gupta et al. 2014). Eighty-nine (89) of the 1224 reported by LASSO were in the downregulated modules, M12 and MOD1. There were significantly more interactions among these 89 genes in STRING analysis (supplementary table S18, Supplementary Material online) than expected (number of edges is 39, expected number is 26; PPI enrichment *P* value: 0.011). Two GO functional ontologies in STRING were also enriched with *P* < 0.01 [GO: 0045202 synapse (13 genes) *P* = 0.0031 (fig. 3); GO: 0044456 synapse part (11 genes) *P* = 0.0071] (supplementary table S15, Supplementary Material online). The intersection of the LASSO male–male list with the set of upregulated genes in ASDs did not show any functional enrichment in STRING. As an example, the SNVs for seven genes from the figure 3, *GABRD, SH2D5*, *GRM8, KCNC3, SYT6, RIMS3*, and *CAP2*, are shown in the supplementary table S19, Supplementary Material online. Some SNVs are overrepresented in ASD patients, but this excess is not overwhelming and significant (supplementary table S19, Supplementary Material online). This tendency seems to create major problems for GWAS-type analyses.

For the LASSO models reported above, table 7 shows the count of the sign of coefficients for the best model produced by glmnet, restricted to those genes that also passed the quality filters described in Materials and Methods. The numbers of genes with positive weights and with negative weights in our LASSO models is roughly balanced (table 7). Thus, at the level of genes, it may be that more efficient translation is preferable for some genes and less efficient translation is preferable for other genes. This balanced result is in contrast to genetic studies seeking rare variants, which have predominantly found likely gene disruptive variants in the heterozygous state. However, it is known that interplay between optimal and suboptimal synonymous codons is extremely complex in eukaryotic and prokaryotic proteins. Although highly expressed genes tend to have an excess of optimal codons, suboptimal codons are functionally important for correct folding of many proteins (Chaney et al. 2017). The importance of both optimal and suboptimal codons for different groups of genes is consistent with a significantly higher fraction of large shifts from optimal codons to suboptimal codons (absolute values of codon shift score ≥0.5) in affected male individuals compared with unaffected male individuals. Moreover, the complementary trend that unaffected male siblings have more large shifts from suboptimal codons to optimal codons than do affected siblings also holds (supplementary table S20, Supplementary Material online).

**Table 7:** Count of the Sign of the Coefficients in the Best Model Generated by Glmnet, for Those Genes that Also Passed Quality Filters

Data Set	Score Used	Positive	Negative	Close to Zero	Total Tested
Ma–Mu	Sum of signs of translation shift scores	597	569	58	16,942
Fa–Fu	Sum of signs of translation shift scores	48	60	5	15,045
Fa–Fu	Count of CpG	84	85	15	12,491

Note.—Models were produced separately for the Ma–Mu and Fa–Fu data sets, using either the sum of signs of the translation shift scores or the count of SNVs in CpG dinucleotides, as indicated in the second column. The “Positive” and “Negative” columns show the count of positive and negative coefficients with absolute value of at least 0.005. The “Close to Zero” column shows the count of coefficients with nonzero, but smaller, absolute value. The “Total Tested” column counts the number of genes considered by glmnet when producing the corresponding model, namely those genes for which at least one individual in the respective data set had a nonzero score.

## Discussion

We previously proposed the hypothesis that perturbations that affect the efficiency of protein translation contribute to the etiology of ASDs (Poliakov et al. 2014). In this study, we confirmed and refined our hypothesis. This hypothesis fits the environment+genetics paradigm because both exposure to toxins and synonymous genetic variations may affect protein translation. Others have previously hypothesized that aberrant translation at synapses is pertinent to the etiology of ASDs (Kelleher and Bear 2008). Translation at synapses is different from translation in other cells, because at synapses, the phenomenon of pausing translation (Buchan and Stansfield 2007; Richter and Coller 2015) is essential to synaptic plasticity (Graber et al. 2013). Darnell and colleagues (Darnell et al. 2011) showed that for hundreds of genes polyribosome stalling is affected by FMRP, the protein that is defective in fragile X syndrome, connecting stalling in translation at synapses explicitly to ASDs and ID. Synonymous substitutions can affect the efficiency of translation and the stability of mRNAs and proteins (Drummond and Wilke 2008; Shabalina et al. 2013; Presnyak et al. 2015). It is generally accepted that translation efficiency is affected by codon usage bias (CUB) via tuning the rate of elongation (Quax et al. 2015). A validated model of codon-tRNA balance for translation efficiency describes how CUB affects translation rates genome-wide, for synonymous mutations (Qian et al. 2012). Furthermore, there are documented cases of different protein structures for the same amino acid sequence translated from two distinct, synonymous nucleotide sequences (Tsai et al. 2008).

We considered the possible interactions with ribosomal profiling scores (significant, at least in male–male pairs) and phylogenetic conservation (not significant). Our pairwise analysis of translation shift scores and ribosome profiling scores for affected and unaffected siblings (tables 2 and 4) suggested that protein dosage effects are likely to be a widespread phenomenon for ASD patients. These effects were most pronounced for male–male comparisons. These results are consistent with the overall trends of translation shift scores (table 1). The *t* test (parametric test) and Wilcoxon matched pairs test (nonparametric test) produced comparable results on most of the tests where we tried both methods. This suggests that results are also consistent from the statistical point of view. We did not apply any corrections for multiple tests, except within the GeneCodis analyses, which do the correction by default.

Recent genomic studies on ASD have focused on whole genome and whole exome sequencing to identify rare variants in genes seen more frequently in ASD subjects than controls (Sanders et al. 2015; Turner et al. 2016; Yuen et al. 2017; Takata et al. 2018). In those studies, pathways of interest are identified in a bottom-up fashion by looking for enrichment of small networks of protein–protein interactions among the proteins encoded by the mutated genes. This approach of looking for rare variants in ASD has been much more successful than genome-wide association studies (GWAS) have been at finding common variants associated with ASD (Wang et al. 2009; Geschwind and State 2015). Yet, multiple modeling studies of ASD inheritance conclude that common variants do contribute significantly to ASD heritability (Gaugler et al. 2014; Robinson et al. 2016). A recent review that tried to compare the different types of evidence suggested that rare variants might explain 21% of ASD (Chaste et al. 2017). Even if one accepts much larger estimates of 40% or more (Ronemus et al. 2014), single rare variants that are associated with ASD do not necessarily cosegregate with disease in multiplex families (Leppa et al. 2016). Moreover, many high-risk rare copy-number variants and other rarer variants are found at measurable frequencies in healthy individuals (Robinson et al. 2016). Some studies of rare variants have found that ASD subjects are more likely than controls to carry multiple rare variants in ASD-associated genes, so that the rare variants confer an additive risk (Buja et al. 2018; Turner et al. 2017). In this view, it is possible that known rare variants and unknown common variants combine additively to confer ASD risk in the same subjects (Weiner et al. 2017).

We suggest that this discrepancy between predictions about the role of common variants and the paucity of GWAS findings can be addressed by looking for large sets of common variants that hint at a mechanism (less translation for males and differential methylation for females). Our methods are fundamentally different from GWAS, which searches for single variants that are statistically associated. One other study tried to look for evidence that de novo synonymous variants with suboptimal codons are associated with ASD (Takata et al. 2016), but was underpowered because of the restriction to de novo variants. We combined the purely mathematical prediction of translation efficiency score with published experimental data on ribosome profiling to validate our mechanistic hypothesis.

Our analysis of ribosome profiling produced better results from the statistical point of view when using brain tumor samples (G14t, supplementary table S9, Supplementary Material online) compared with normal brain samples (G14n, table 4). This may be a result of disturbed translation in both tumor cells (Gonzalez et al. 2014) and ASD patients (Kelleher and Bear 2008). An alternative explanation is that there may have been better sampling of reads from tumor cells. The disturbed translation was suggested to be an important hallmark of ASD (Kelleher and Bear 2008).

We also did an analysis of SNVs at CpG dinucleotides, which suggested that female ASD patients have a significantly higher frequency of SNVs in these dinucleotides, whereas no similar tendencies were found for male–male pairs. CpG dinucleotides can be methylated to form 5-methylcytosine. In mammals, methylating the cytosine within a gene can substantially change its expression. Monogenic, syndromic forms of autism that involve methylation defects, including Rett syndrome, Prader–Willi and Angelman syndromes, and others, suggested that differential methylation of genes may underlie one aspect of ASD pathogenesis (Vogel Ciernia and LaSalle 2016). Moreover, several studies of likely deleterious mutations and pathway enrichment have observed that genes controlling chromatin accessibility or remodeling (and hence gene expression) are enriched for genes with recurrent mutations (Geschwind and State 2015; Sanders et al. 2015; Geisheker et al. 2017). The observed tendencies may reflect such effects in gene expression triggered by environmental factors. The combination of our results using ribosome profiling and CpG dinucleotides confirms that gender-specific properties of ASD are important (Frazier et al. 2014; Duvekot et al. 2017), and suggests some differences in genetic mechanisms for ASD susceptibility in the two genders.

We found dramatic differences between the lists of the merged essential+nonessential gene set and all other unclassified human genes (supplementary table S8, Supplementary Material online). We conjecture that this distinction reflects a protein dosage effect in the classification itself, namely that genes with moderate to high expression in the brain are substantially enriched in the merged essential+nonessential gene set, whereas genes with low or no expression are mostly unclassified.

Delineation of specific genes associated with ASD is a frequent approach to studying this complex disease (Sanders et al. 2015; Yuen et al. 2017). One of the most widely used lists is the SFARI list (https://gene.sfari.org/). Our lists of genes associated with ASD were generated using LASSO analysis for translation shift score for male–male and female–female patient-sibling pairs and for CpG containing SNVs for female–female patient-sibling pairs. We found several promising candidate pathways. The Wnt signaling pathway has been implicated in ASD by several studies (Caracci et al. 2016; de la Torre-Ubieta et al. 2016; Kwan et al. 2016; Takata et al. 2016). This pathway seems central for synapse formation/plasticity as well as for cancer initiation/progression (Anastas and Moon 2013; Caracci et al. 2016). We also found that SNVs in downregulated coexpressed of proteins from ASD patients are enriched in synaptic proteins. We also found that predicted lists of genes are highly enriched in ATP-binding and nucleotide binding proteins per GO ontologies. One recent hypothesis of autism etiology implicates ATP as a central stress signaling molecule in the cell danger response (CDR) response, (Naviaux 2014; Naviaux et al. 2013, 2017). Thus, enrichment of ATP binding and ATPase activity in male–male pairs is in an agreement with this hypothesis All these findings confirm that ASD etiology is extremely complex and likely to require larger sets of affected families for more detailed studies of ASD.

Towards this objective, public availability of large data sets via repositories such as NDAR and MSSNG is essential to allowing more researchers to participate in the search for factors that contribute to ASDs. Our findings support the work of other researchers who have suggested that Wnt signaling and ATP/ATP-ase activities may play mechanistic roles in the causes of autism. Our significant findings about translation shift scores support the general theory that environmental toxins may combine with genetic variation to impact the translation efficiency of hundreds of brain-expressed genes, thereby affecting disease propensity. In light of the accumulating evidence both genetic and environmental factors in ASD susceptibility, it is essential to search for gene-environment interactions, but designing such studies is very difficult (Kim and Leventhal 2015). It is an interesting challenge, not just for autism, to develop new methods to study the efficiency of protein translation genome-wide.

Whether allele frequencies in ASD-related SNVs have changed in conjunction with the increase in ASD prevalence is an open question (Polimanti and Gelernter 2017). It has been suggested that at least two different evolutionary mechanisms appear to be present in relation to ASD genetics: 1) rare disruptive alleles eliminated by purifying selection and 2) common alleles selected for their beneficial effects on cognitive skills (Polimanti and Gelernter 2017). This combination of mechanisms would explain part of the increase in ASD prevalence, which is quite unexpected for a trait being selected against. At least the forms of autism that include ID would be expected to be selected against. From this evolutionary perspective, the changes in allele frequencies and increase in ASD prevalence could be evolutionary costs of polygenic adaptation related to cognitive ability (Polimanti and Gelernter 2017).

Our results are consistent with the hypothesis that allele frequency changes are subtle. Hence, the statistical signals can be detected more effectively by analyzing many variants at once rather than analyzing one variant at a time in the GWAS paradigm. Human adaptation in response to the selection of polygenic phenotypes due to short-term environmental factors may occur via subtle allele frequency shifts at many loci (Chaste et al. 2017; Turner et al. 2017; Weiner et al. 2017).

## Supplementary Material

Supplementary data are available at *Genome Biology and Evolution* online.

## Supplementary Material

Supplementary Data
