# Supplementary material for: Genome-Wide Changes in Protein Translation Efficiency Are Associated with Autism
Source: Genome Biol Evol. 2018 Jul 7;10(8):1902–19. doi: 10.1093/gbe/evy146 (PMC6086092; doi:10.1093/gbe/evy146)
Supplement: Supplementary Data [file evy146_supp.zip › Supplementary_Tables_resubmission_20180604fixed.docx]

## Supplementary Tables

Supplementary Table 1. Codon usage frequencies from brain-specific genes. For each codon, the number immediately to its right is the number of occurrences of this codon per 1000bp.

| TTT | 15.1 | CTT | 10.0 | ATT | 11.8 | GTT | 7.5 |
| --- | --- | --- | --- | --- | --- | --- | --- |
| TTC | 24.0 | CTC | 24.9 | ATC | 24.0 | GTC | 16.9 |
| TTA | 4.8 | CTA | 5.6 | ATA | 5.0 | GTA | 5.3 |
| TTG | 9.7 | CTG | 46.5 | ATG | 21.3 | GTG | 30.9 |
| TCT | 13.0 | CCT | 18.1 | ACT | 10.6 | GCT | 16.3 |
| TCC | 21.1 | CCC | 27.4 | ACC | 21.7 | GCC | 35.5 |
| TCA | 10.0 | CCA | 16.2 | ACA | 12.3 | GCA | 13.3 |
| TCG | 6.5 | CCG | 11.0 | ACG | 8.2 | GCG | 11.0 |
| TAT | 8.9 | CAT | 8.0 | AAT | 11.7 | GAT | 15.5 |
| TAC | 16.1 | CAA | 18.5 | AAC | 19.1 | GAC | 26.7 |
| TAA | 0.4 | CAG | 8.2 | AAA | 16.0 | GAA | 18.7 |
| TAG | 0.5 | CAC | 32.8 | AAG | 30.1 | GAG | 41.2 |
| TGT | 9.7 | CGT | 3.9 | AGT | 9.8 | GGT | 9.3 |
| TGC | 15.5 | CGC | 14.5 | AGC | 23.7 | GGC | 30.5 |
| TGA | 0.9 | CGA | 5.2 | AGA | 8.9 | GGA | 14,1 |
| TGG | 14.0 | CGG | 13.8 | AGG | 13.8 | GGG | 21.3 |

Supplementary Table S2. List of ribosome profiling sets used in this study. SRA is the Short Read Archive at the National Center for Biotechnology Information.

| Description | Short name | SRA source |
| --- | --- | --- |
| Normal brain samples | G14n | SRR1562539 |
| Tumor brain samples | G14t | SRR1562539 |
| Treated cycloheximide HEK293 | L12 | SRR618771 |
| Treated cycloheximide HeLa | S11 | SRR407636 |
| Normal mito samples | R13 | SRR935448 |

|  |  |  |
| --- | --- | --- |

Supplementary Table S3. The number of SNVs (rare variants) that have positive (POS) and negative (NEG) values of the translation shift score. In this analysis, we tested the effect of changing the threshold for rare variants from the baseline MAF < 10%.

Affected Unaffected

#Families POS NEG POS NEG P_Fisher_

5% threshold for the MAF of rare SNVs

**All 696128 538418 695633 541399 0.0075**

**Ma-Mu 280788 221046 280358 222463 0.0244**

Ma-Fu 323692 251962 323037 252858 0.0691

**Fa-Mu 44073 33919 44074 34569 0.0317**

Fa-Fu 47575 31491 48164 31509 0.8746

15% threshold for the MAF of rare SNVs

**All 1394532 1028954 1393807 1037651 5.66 x 10^-7^**

**Ma-Mu 566220 418517 564155 420891 0.0006**

**Ma-Fu 645639 473556 647184 479369 0.0001**

**Fa-Mu 88473 66139 87175 66448 0.0038**

Fa-Fu 94200 70742 95293 70943 0.8928

One-tail Fisher exact tests (http://www.langsrud.com/fisher.htm) were used to test whether rare SNVs in affected individuals tend to have relatively more SNVs with a positive shift than unaffected individuals. Ma-Mu is affected male-unaffected male siblings, Ma-Fu is affected male - unaffected female siblings, Fa-Mu is affected female - unaffected male siblings, Fa-Fu is affected female - unaffected female siblings. Significant deviations according to the Fisher exact test from the homogeneous 2x2 tables are bold and underlined.

Supplementary Table S4. Differences between affected and unaffected siblings using median translation shift scores calculated in each individual (synonymous SNVs with absolute values of codon shift score ≥ 0.25/0.75 and all non-synonymous SNVs).

The threshold for synonymous SNVs = 0.25

Affected Unaffected Paired *t*-test Paired Wilcoxon Z

Mean SD Mean SD (P-value) (P-value)

All 0.119 0.043 0.115 0.044 **3.1 (0.0021) 2.9 (0.0036)**

Ma-Mu 0.120 0.044 0.113 0.044 **3.0 (0.0024) 3.2 (0.0014)**

Ma-Fu 0.119 0.040 0.117 0.043 0.8 (0.4101) 0.5 (0.6224)

Fa-Mu 0.123 0.039 0.115 0.041 1.9 (0.0564) **2.0 (0.0498)**

Fa-Fu 0.111 0.053 0.109 0.048 0.2 (0.8198) 0.7 (0.9475)

The threshold synonymous SNVs = 0.75

All -0.024 0.035 -0.029 0.036 **4.1 (4x10^-5^) 1.9 (7x10^-5^)**

Ma-Mu -0.026 0.035 -0.031 0.035 **2.7 (0.0074) 2.6 (0.0095)**

Ma-Fu -0.022 0.034 -0.028 0.036 **3.5 (0.0005) 3.4 (0.0006)**

Fa-Mu -0.019 0.037 -0.023 0.035 0.7 (0.4578) 0.5 (0.6392)

Fa-Fu -0.036 0.035 -0.033 0.035 -0.5 (0.5266) 0.4 (0.7064)

Two-tailed paired tests were used to compare median values of translation shift scores calculated in each individual. Ma-Mu is affected male-unaffected male siblings, Ma-Fu is affected male - unaffected female siblings, Fa-Mu is affected female - unaffected male siblings, Fa-Fu is affected female - unaffected female siblings. Codon usage frequencies were taken from Semon et al. (Semon, et al. 2006), as used in (Poliakov, et al. 2014).

Supplementary Table S5. Differences between affected and unaffected siblings using median translation shift scores calculated in each individual for different codon usage tables.

Affected Unaffected Paired *t*-test Paired Wilcoxon Z

Mean SD Mean SD (P-value) (P-value)

Codon frequencies from ([Plotkin, Robins et al. 2004](#_ENREF_63))

All 0.024 0.029 0.022 0.028 **2.4 (0.01652)** **2.2 (0.02804)**

Ma-Mu 0.024 0.028 0.020 0.027 **2.8 (0.00492)** **2.7 (0.00603)**

Ma-Fu 0.023 0.028 0.024 0.029 -0.8 (0.42378) 1.0 (0.30456)

Fa-Mu 0.030 0.028 0.019 0.022 **3.1 (0.00250)** **3.1 (0.00229)**

Fa-Fu 0.022 0.033 0.018 0.026 0.9 (0.39189) 0.3 (0.77714)

Codon frequencies averaged over all human genes ([Warrington, Nair et al. 2000](#_ENREF_94))

All 0.017 0.037 0.013 0.037 **2.9 (0.00420)** **2.8 (0.00464)**

Ma-Mu 0.017 0.038 0.011 0.037 **2.8 (0.00557)** **2.8 (0.00490)**

Ma-Fu 0.016 0.036 0.016 0.038 -0.1 (0.89990) 0.2 (0.87681)

Fa-Mu 0.027 0.036 0.010 0.032 **3.8 (0.00022)** **3.6 (0.00028)**

Fa-Fu 0.014 0.037 0.009 0.037 1.0 (0.30279) 0.8 (0.41376)

Codon frequencies averaged over high confidence SFARI genes

All 0.043 0.038 0.041 0.037 **2.1 (0.03415) 2.1 (0.03444)**

Ma-Mu 0.044 0.039 0.039 0.037 **2.6 (0.00847)** **2.4 (0.01549)**

Ma-Fu 0.041 0.037 0.043 0.038 -1.3 (0.18926) 1.1 (0.26010)

Fa-Mu 0.052 0.039 0.038 0.035 **3.2 (0.00201) 3.0 (0.00302)**

Fa-Fu 0.040 0.037 0.037 0.037 0.4 (0.65280) 0.4 (0.70638)

Codon frequencies averaged over ASD susceptibility genes genes from (Poliakov, et al. 2014)

All 0.079 0.052 0.075 0.051 **2.4 (0.01863) 2.7 (0.00718)**

Ma-Mu 0.081 0.051 0.072 0.052 **3.3 (0.00097)** **3.6 (0.00030)**

Ma-Fu 0.079 0.050 0.079 0.051 -0.4 (0.96431) 0.1 (0.88594)

Fa-Mu 0.089 0.043 0.078 0.041 **2.2 (0.02984) 2.0 (0.04732)**

Fa-Fu 0.065 0.073 0.070 0.053 -0.6 (0.56398) 0.3 (0.76117)

Two-tailed paired tests were used to compare values of translation shift scores calculated in each individual. Ma-Mu is affected male-unaffected male siblings, Ma-Fu is affected male - unaffected female siblings, Fa-Mu is affected female - unaffected male siblings, Fa-Fu is affected female - unaffected female siblings. SFARI codon frequencies were extracted from the list of high confidence SFARI genes (March 14, 2018, https://www.sfari.org/resource/sfari-gene/) (*ADNP, ANK2, ARID1B, ASH1L, ASXL3, CHD8, CUL3, DSCAM. DYRK1A, GRIN2B, KATNAL2, KMT2A, KMT5B, MYTIL, NAA15, POGZ, PTEN, RELN, SCN2A, SETD5, SHANK3, SYNGAP1, TBR1, TRIP12*). The list of ASD susceptibility genes genes studied in (Poliakov, et al. 2014) was: *NRXN1, NLGN3, MECP2, SHANK2, SHANK3, AUTS2, CADPS2, FOXP2, RBFOX1, GRM1, GRM5, HRAS, MAP2K1, MAP2K2, PIK3CA, TSC1, TSC2, UBE3A, GRIN2A , GRIN2B , GRIN2C , GRIN2D , GRIN3B*.

Supplementary Table S6. Differences between affected and unaffected siblings using the total number of SNVs calculated in each individual.

Affected Unaffected Paired *t*-test Paired Wilcoxon Z

Mean SD Mean SD (P-value) (P-value)

All 1038 258 1042 262 -1.8 (0.0674) 1.9 (0.0623)

Ma-Mu 1038 251 1037 244 0.1 (0.9078) 0.3 (0.9767)

Ma-Fu 1042 248 1053 252 -**3.2 (0.0014) 4.2 (2x10^-5^)**

Fa-Mu 1042 262 1038 258 1.8 (0.0673) **2.3 (0.0174)**

Fa-Fu 1045 318 1056 306 -1.3 (0.2050) 1.2 (0.2320)

Two-tailed paired tests were used to compare the total number of SNVs calculated in each individual. Ma-Mu is affected male-unaffected male siblings, Ma-Fu is affected male - unaffected female siblings, Fa-Mu is affected female - unaffected male siblings, Fa-Fu is affected female - unaffected female siblings.

Supplementary Table S7. Differences between affected and unaffected siblings using median translation shift scores calculated in each individual for the SFARI list of genes.

Affected Unaffected Paired *t*-test Paired Wilcoxon Z

Mean SD Mean SD (P-value) (P-value)

All 0.034 0.164 0.026 0.167 1.5 (0.1372) 1.9 (0.0587)

Ma-Mu 0.026 0.17 0.007 0.179 **2.2 (0.0302) 2.6 (0.0103)**

Ma-Fu 0.042 0.158 0.038 0.157 0.6 (0.5362) 0.8 (0.4349)

Fa-Mu 0.064 0.158 0.055 0.157 0.4 (0.6841) 0.3 (0.7997)

Fa-Fu 0.001 0.163 0.04 0.161 -1.8 (0. 0706) 1.5 (0.1319)

Two-tail tests were used to compare values of translation shift scores calculated in each individual. Ma-Mu is affected male-unaffected male siblings, Ma-Fu is affected male - unaffected female siblings, Fa-Mu is affected female - unaffected male siblings, Fa-Fu is affected female - unaffected female siblings.

Supplementary Table S8. Differences between affected and unaffected siblings using median translation shift scores calculated in each individual for essential (EGenes) and nonessential (NEGenes) genes.

Affected Unaffected Paired *t*-test Paired Wilcoxon Z

Mean SD Mean SD (P-value) (P-value)

All EGenes 0.037 0.084 0.029 0.085 **3.0 (0.0024) 2.9 (0.0038)**

NEGenes 0.023 0.072 0.016 0.073 **2.9 (0.0035)** **3.0 (0.002)**

EGenes+NEGenes 0.026 0.059 0.019 0.058 **4.0 (6x10^-5^) 4.1 (4x10^-5^)**

Unclassified genes 0.002 0.049 -0.002 0.050 2.0 (0.0511) 1.8 (0.0799)

Ma-Mu EGenes 0.036 0.083 0.025 0.086 **2.6 (0.0091) 2.5 (0.0132)**

NEGenes 0.023 0.072 0.014 0.074 **2.5 (0.0119) 2.7 (0.0071)**

EGenes+NEGenes 0.024 0.057 0.015 0.058 **3.7 (0.0002) 3.8 (0.0001)**

Unclassified genes 0.003 0.051 -0.001 0.052 0.6 (0.5798) 0.4 (0.7184)

Ma-Fu EGenes 0.039 0.084 0.034 0.081 1.4 (0.1731) 1.5 (0.1456)

NEGenes 0.024 0.072 0.017 0.071 **2.1 (0.0327) 2.3 (0.0214)**

EGenes+NEGenes 0.028 0.055 0.022 0.058 **2.5 (0.0145) 2.4 (0.0169)**

Unclassified genes 0.003 0.048 -0.001 0.050 1.7 (0.0879) 1.4 (0.1513)

Fa-Mu EGenes 0.057 0.075 0.042 0.078 1.5 (0.1499) 1.1 (0.278)

NEGenes 0.025 0.068 0.020 0.068 0.5 (0.6197) 0.2 (0.878)

EGenes+NEGenes 0.034 0.060 0.025 0.057 1.2 (0.2313) 0.7 (0.4636)

Unclassified genes 0.011 0.049 0.001 0.045 1.6 (0.1063) **2.2 (0.0253)**

Fa-Fu EGenes 0.016 0.091 0.012 0.108 0.4 (0.7016) 0.2 (0.8614)

NEGenes 0.017 0.079 0.024 0.079 -0.1 (0.4605) 0.8 (0.4375)

EGenes+NEGenes 0.011 0.082 0.017 0.017 -0.7 (0.4815) 0.7 (0.5060)

Unclassified genes -0.009 0.047 -0.011 0.049 0.4 (0.7170) 0.3 (0.7360)

Two-tail tests were used to compare values of translation shift scores calculated in each individual. Ma-Mu is affected male-unaffected male siblings, Ma-Fu is affected male - unaffected female siblings, Fa-Mu is affected female - unaffected male siblings, Fa-Fu is affected female - unaffected female siblings. ”EGenes+NEGenes” stands for a merged EGenes and NEGenes gene set, “Unclassified genes” stands for all other human genes.

Supplementary Table S9. Differences between affected and unaffected siblings using median ribosome profiling scores for various samples calculated in each individual.

Affected Unaffected Paired *t*-test Paired Wilcoxon Z

Mean SD Mean SD (P-value) (P-value)

Dataset G14t

All 1.49 1.07 1.31 1.16 **4.7 (0.000003)** **4.4 (0.00001)**

Ma-Mu 1.4 1.1 1.2 1.24 **3.4 (0.00066)** **3.1 (0.00203)**

Ma-Fu 1.57 1.0 1.43 1.11 **2.8 (0.005034) 2.7 (0.006512)**

Fa-Mu 1.65 1.0 1.48 1.01 1.3 (0.196696) 1.1 (0.289713)

Fa-Fu 1.22 1.32 1.08 1.1 0.9 (0.386836) 1.1 (0.269636)

Dataset L12

All 0.7 0.72 0.63 0.78 **2.8 (0.00534)** **2.4 (0.01587)**

Ma-Mu 0.67 0.74 0.62 0.78 1.2 (0.21222) 1.1 (0.27919)

Ma-Fu 0.73 0.68 0.62 0.78 **3.1 (0.00207) 2.7 (0.00771)**

Fa-Mu 0.7 0.72 0.73 0.68 -0.4 (0.65688) 0.4 (0.69501)

Fa-Fu 0.59 0.81 0.59 0.81 0. (1.) 0. (1.)

Dataset S11

All 0.7 0.71 0.6 0.8 **4.1 (0.00004)** **3.5 (0.00043)**

Ma-Mu 0.69 0.73 0.58 0.81 **2.7 (0.00663)** **2.4 (0.01876)**

Ma-Fu 0.74 0.67 0.62 0.78 **3.8 (0.00011) 2.9 (0.00427)**

Fa-Mu 0.75 0.66 0.7 0.72 0.7 (0.51529) 0.6 (0.56631)

Fa-Fu 0.53 0.85 0.51 0.86 0.1 (0.90332) 0. (1.)

Dataset R13

All 0.82 0.72 0.73 0.78 **4.0 (0.000064)** **4.8 (0.000001)**

Ma-Mu 0.81 0.73 0.73 0.78 **2.0 (0.047584)** **2.5 (0.011263)**

Ma-Fu 0.86 0.68 0.72 0.78 **4.1 (0.000032)** **4.5 (0.000006)**

Fa-Mu 0.77 0.78 0.81 0.71 -0.5 (0.642498) 0.1 (0.926732)

Fa-Fu 0.69 0.84 0.67 0.84 0.1 (0.898403) 0.6 (0.563132)

Two-tail tests were used to compare values of signed ribosome profiling scores calculated in each individual. Ma-Mu is affected male-unaffected male siblings, Ma-Fu is affected male - unaffected female siblings, Fa-Mu is affected female - unaffected male siblings, Fa-Fu is affected female - unaffected female siblings.

Supplementary Table S10. Differences between affected and unaffected siblings using median modified ribosome profiling scores for various samples calculated in each individual.

Affected Unaffected Paired *t*-test Paired Wilcoxon Z

Mean SD Mean SD (P-value) (P-value)

Modified ribosomal profiling scores calculated for SNV-affected codons

Dataset G14n

All 1.25 1.42 1.06 1.45 **4.2 (0.00003)** **4.1 (0.00004)**

Ma-Mu 1.15 1.21 1.06 1.18 1.5 (0.13395) 1.6 (0.11005)

Ma-Fu 1.37 1.40 1.13 1.42 **3.5 (0.00046) 3.5 (0.00051)**

Fa-Mu 1.36 1.39 1.13 1.30 1.5 (0.14374) 1.4 (0.17106)

Fa-Fu 1.01 1.49 0.93 1.55 0.4 (0.66218) 0.3 (0.74579)

Dataset G14t

All 1.73 1.25 1.65 1.28 1.9 (0.05585) **2.1 (0.03353)**

Ma-Mu 1.66 1.33 1.68 1.28 -0.3 (0.75100) 0.3 (0.74344)

Ma-Fu 1.63 1.37 1.45 1.43 **2.8 (0.00596) 2.8 (0.00512)**

Fa-Mu 1.68 1.23 1.31 1.47 1.9 (0.05389) **2.0 (0.04773)**

Fa-Fu 1.62 1.50 1.61 1.70 0. (0.98068) 0.3 (0.74499)

Modified ribosomal profiling scores calculated for SNV-affected codons multiplied by translation shift scores

Dataset G14n

All 0.30 0.31 0.27 0.32 **2.7 (0.006196)** **2.6 (0.008086)**

Ma-Mu 0.30 0.30 0.26 0.33 **2.1 (0.033885)** **2.1 (0.03589)**

Ma-Fu 0.31 0.29 0.29 0.31 1.0 (0.302985) 0.8 (0.431749)

Fa-Mu 0.36 0.32 0.25 0.26 **3.1 (0.002131) 2.8 (0.005956)**

Fa-Fu 0.27 0.37 0.26 0.35 0.1 (0.916534) 0.4 (0.712435)

Dataset G14t

All 0.28 0.45 0.26 0.41 1.7 (0.081876) **2.6 (0.009144)**

Ma-Mu 0.26 0.44 0.26 0.40 0.0 (0.971552) 0.6 (0.575796)

Ma-Fu 0.31 0.40 0.26 0.40 **2.7 (0.007783) 2.7 (0.006638)**

Fa-Mu 0.33 0.34 0.23 0.46 1.8 (0.088449) 1.4 (0.173258)

Fa-Fu 0.16 0.74 0.21 0.49 -0.5 (0.617554) 0.5 (0.651416)

Two-tail tests were used to compare values of modified ribosome profiling scores calculated in each individual. Ma-Mu is affected male-unaffected male siblings, Ma-Fu is affected male - unaffected female siblings, Fa-Mu is affected female - unaffected male siblings, Fa-Fu is affected female - unaffected female siblings.

Supplementary Table S15. STRING gene enrichment analysis. No significant enrichment was found for the GO categories ‘Molecular Function’ or ‘Biological Process’ among the Ma-Mu downregulated genes.

| **Ma-Mu all genes** | | | |
| --- | --- | --- | --- |
| **Pathway ID** | Pathway description | Genes | False discovery rate |
| **Molecular Function** | | | |
| **GO:0016787** | Hydrolase activity | 180 | 0.0027 |
| **GO:0003824** | Catalytic activity | 355 | 0.00835 |
| **GO:0042623** | ATPase activity, coupled | 32 | 0.0356 |
| **Biological Process** | | | |
| **GO:0008152** | Metabolic process | 585 | 0.00156 |
| **GO:0044238** | Primary metabolic process | 530 | 0.00156 |
| **GO:0071704** | Organic substance metabolic process | 536 | 0.00481 |
| **GO:0044237** | Cellular metabolic process | 513 | 0.0163 |
| **GO:0009987** | Cellular process | 712 | 0.0435 |
| **Cellular component** | | | |
| **GO:0043231** | intracellular membrane-bounded organelle | 654 | 0.000104 |
| **GO:0043229** | intracellular organelle | 690 | 0.000347 |
| **GO:0043227** | membrane-bounded organelle | 705 | 0.000402 |
| **GO:0044424** | intracellular part | 759 | 0.000539 |
| **GO:0043226** | organelle | 733 | 0.000569 |
| **GO:0005622** | intracellular | 771 | 0.00154 |
| **GO:0005737** | cytoplasm | 619 | 0.00154 |
| **GO:0044444** | cytoplasmic part | 466 | 0.00453 |
| **GO:0005606** | laminin-1 complex | 3 | 0.0347 |
| **GO:0044422** | organelle part | 483 | 0.0347 |
| **Ma-Mu downregulated genes** | | | |
| **Cellular Component** | | | |
| **GO:0045202** | Synapse | 13 | 0.00306 |
| **GO:0044456** | Synapse part | 11 | 0.00711 |
| **GO:0005737** | Cytoplasm | 59 | 0.0122 |
| **GO:0098793** | Presynapse | 6 | 0.0122 |
| **GO:0044444** | Cytoplasmic part | 47 | 0.0232 |
| **GO:0008021** | Synaptic vesicle | 5 | 0.0469 |

Supplementary Table S18. STRING network statistics.

| **Gene List** | **Nodes** | **Edges** | **Mean Node Degree** | **Avg. Clustering Coefficient** | **Expected Nodes** | **PPI Enrichment P-value** |
| --- | --- | --- | --- | --- | --- | --- |
| **Ma-Mu translation shift score list** | 1221 | 3809 | 6.24 | 0.384 | 3826 | 0.613 |
| **Fa-Fu translation shift score list** | 112 | 22 | 0.393 | 0.156 | 25 | 0.726 |
| **Fa-Fu CpG list** | 183 | 25 | 0.273 | 0.182 | 25 | 0.497 |
| **Intersection of LASSO male-male list and downregulated modules (M12 and MOD1)** | 89 | 39 | 0.876 | 0.287 | 26 | 0.0106 |

Ma-Mu is affected male-unaffected male siblings, Fa-Fu is affected female - unaffected female siblings.

Supplementary Table S19. List of analyzed SNVs for *GABRD,* *SH2D5*, *GRM8, KCNC3, SYT6, RIMS3,* and *CAP2* genes for male-male (Ma-Mu) pairs.

**SNV Position Chromosome #SNVs in #SNVs in**

***GABRG* affected unaffected**

GGT>GGC 1957037 1 237 228

AGC>AGT 1960674 1 93 83

GCC>GCT 1961466 1 27 13

CGC>CAC 1959699 1 10 14

ACG>ACA 1957121 1 2 1

CAC>CAT 1957112 1 2 1

AAC>AAT 1961144 1 1 1

***SH2D5***

CAG>CAA 21050958 1 169 171

GGC>AGC 21050969 1 1 1

***GRM8***

AGA>CGA 126173755 7 11 3

TTC>TTT 126882902 7 2 5

CCG>GCG 126173902 7 1 1

GGT>GGC 126746584 7 2 1

ATC>ATT 126249446 7 1 1

***KCNC3***

GCC>GCT 50826995 19 4 1

TAC>TAT 50826806 19 3 1

TCG>TCA 50826569 19 4 8

***SYT6***

TCG>TCA 114680540 1 142 138

ACC>ATC 114680310 1 99 104

CGT>CAT 114680442 1 1 1

TAC>TAT 114680471 1 1 1

ACT>ACA 114646293 1 3 1

TAT>TAC 114641880 1 2 1

AAC>AAT 114680315 1 1 1

***RIMS3***

GGC>AGC 41094496 1 1 1

ACC>ACA 41094554 1 3 1

CCA>GCA 41094964 1 1 3

TCA>TCC 41107568 1 1 3

***CAP2***

AAA>AAG 17543302 6 238 230

TTT>TTC 17426846 6 6 5

TAT>TGT 17541324 6 17 9

CAT>CAC 17541340 6 8 2

ACA>GCA 17541308 6 3 1

ATA>ATC 17543187 6 5 7

ATC>ATT 17551781 6 1 4

CCC>ACC 17541305 6 2 7

Supplementary Table S20. The number of synonymous SNVs (rare variants) that have positive (POS) and negative (NEG) values of the translation shift score (TSS) for male-male pairs. In this analysis, we tested the effect of splitting rare variants (MAF < 10%) into large (absolute values of codon shift score ≥ 0.5) and small shifts (absolute values of codon shift score < 0.5) in TSS.

POS TSS NEG TSS

Affected Unaffected Affected Unaffected

Large 114246 | 113122 92353 | 92696

Small 100206 | 100452 32523 | 32013

Ratio 1.14 1.13 2.84 2.90

Probability P_pos_=0.02215 P_neg_=0.01163

(Fisher

exact test)

The raw data were taken from the analysis described in Table 1 (Ma-Mu, All synonymous SNVs).
