# Supplementary figures and images for: Genome-Wide Changes in Protein Translation Efficiency Are Associated with Autism
Source: Genome Biol Evol. 2018 Jul 7;10(8):1902–19. doi: 10.1093/gbe/evy146 (PMC6086092; doi:10.1093/gbe/evy146)

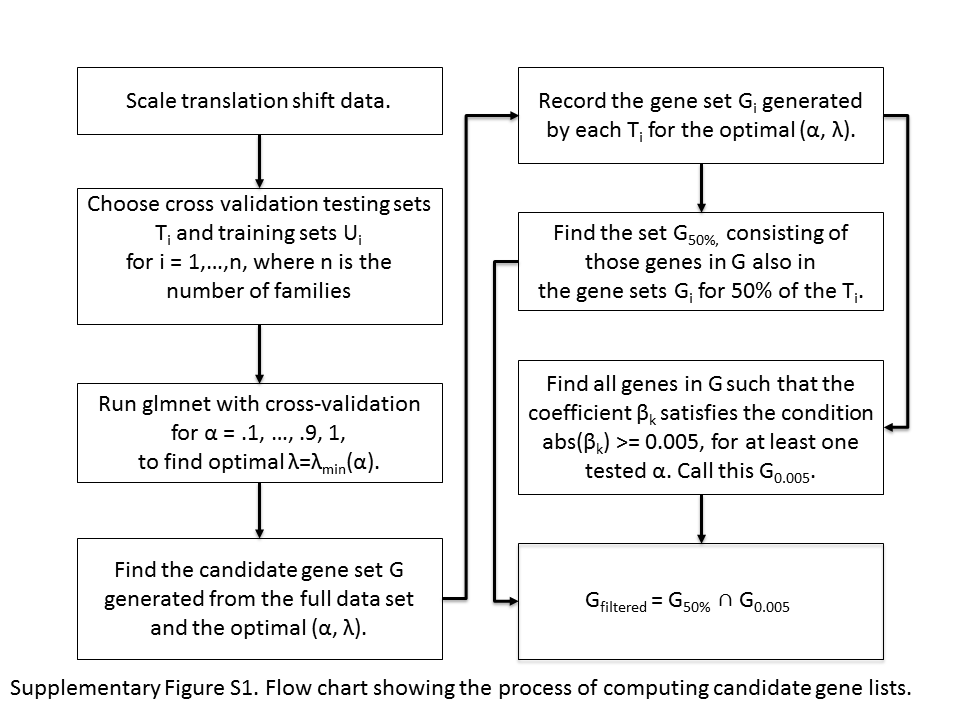

Supplement: Supplementary Data [file evy146_supp.zip › S1_Fig_S1.tif]
